# A graph neural network approach for molecule carcinogenicity prediction

**DOI:** 10.1093/bioinformatics/btac266

**Published:** 2022-06-27

**Authors:** Philip Fradkin, Adamo Young, Lazar Atanackovic, Brendan Frey, Leo J Lee, Bo Wang

**Affiliations:** Department of Electrical & Computer Engineering, University of Toronto, Toronto, ON M5S 3G8, Canada; Vector Institute, Toronto, ON M5G 1M1, Canada; Vector Institute, Toronto, ON M5G 1M1, Canada; Department of Computer Science, University of Toronto, Toronto, ON M5S 2E4, Canada; Department of Electrical & Computer Engineering, University of Toronto, Toronto, ON M5S 3G8, Canada; Vector Institute, Toronto, ON M5G 1M1, Canada; Department of Electrical & Computer Engineering, University of Toronto, Toronto, ON M5S 3G8, Canada; Vector Institute, Toronto, ON M5G 1M1, Canada; Department of Computer Science, University of Toronto, Toronto, ON M5S 2E4, Canada; Department of Electrical & Computer Engineering, University of Toronto, Toronto, ON M5S 3G8, Canada; Vector Institute, Toronto, ON M5G 1M1, Canada; Vector Institute, Toronto, ON M5G 1M1, Canada; Department of Computer Science, University of Toronto, Toronto, ON M5S 2E4, Canada; Laboratory Medicine and Pathobiology, University of Toronto, Toronto, ON M5S 1A8, Canada; Peter Munk Cardiac Center, UHN, Toronto, ON M5G 2N2, Canada

## Abstract

**Motivation:**

Molecular carcinogenicity is a preventable cause of cancer, but systematically identifying carcinogenic compounds, which involves performing experiments on animal models, is expensive, time consuming and low throughput. As a result, carcinogenicity information is limited and building data-driven models with good prediction accuracy remains a major challenge.

**Results:**

In this work, we propose CONCERTO, a deep learning model that uses a graph transformer in conjunction with a molecular fingerprint representation for carcinogenicity prediction from molecular structure. Special efforts have been made to overcome the data size constraint, such as multi-round pre-training on related but lower quality mutagenicity data, and transfer learning from a large self-supervised model. Extensive experiments demonstrate that our model performs well and can generalize to external validation sets. CONCERTO could be useful for guiding future carcinogenicity experiments and provide insight into the molecular basis of carcinogenicity.

**Availability and implementation:**

The code and data underlying this article are available on github at https://github.com/bowang-lab/CONCERTO

## 1 Introduction

Globally, cancer is the second leading cause of death, and accurate molecule carcinogenicity prediction holds promise in decreasing the likelihood of disease onset (Roser and Ritchie, 2015). Cancer can be broken down by onset cause: random somatic mutations during cell division, exposure to harmful radiation or molecule reactivity with DNA (Balmain, 2020). Two major methods for measuring chemical carcinogenesis potential are carcinogenic and mutagenic experiments (Jacobs and Brown, 2015; Smietana *et al.*, 2016). Carcinogenic data is considered more accurate as it directly measures tumor growth in animals; however, experiments can be costly and time consuming. In addition it is able to capture compound carcinogenicity acting through DNA reactivity (genotoxic effects) and mis-regulation of cell function (non-genotoxic effects) (Wolf *et al.*, 2019). In contrast, mutagenicity experiments are conducted in bacterial cultures and tend to be significantly cheaper and faster, but result in higher rates of false positives and capture only genotoxic effects (Walmsley and Billinton, 2011). *In silico* predictions to assess carcinogenicity provide an appealing alternative, and can help select compounds for costly downstream analysis.

Traditionally, carcinogenic compounds have been identified as a result of observational studies in sub-populations with increased cancer penetrance. This approach is effective in two scenarios: the first is if the compound is extremely carcinogenic, e.g. aristolochic acid, which was present in certain herbal supplements before being identified as one of the most potent compounds in the carcinogenic potency database (CPDB) (Gold *et al.*, 2005). The second is when a large enough sub-population is repeatedly exposed to a moderately carcinogenic compound, as was the case with chimney sweepers in 18th century England, whose exposure to soot was correctly identified by Percivall Pott in 1775 as a source of illness (Waldron, 1983). These approaches cannot identify chemicals of intermediate potency and prevalence, leading to continued exposure of the population to unidentified carcinogens. A high throughput computational method for predicting molecular carcinogenicity can provide a filter for discovering high likelihood carcinogenic compounds that would be otherwise missed by traditional identification workflows.

Current state-of-the-art solutions for molecule carcinogenicity prediction train models using ASCII string representations of molecules through simplified molecular-input line-entry systems (SMILES) or molecular fingerprints, hand engineered features capturing core molecular properties (Wang *et al.*, 2020; Weininger, 1988; Zhang *et al.*, 2017). A major drawback of these approaches is that the model architecture does not make use of the topology of the molecular graph. Graph neural networks (GNNs) are invariant to the ordering of atoms in a molecular graph and can leverage their respective node and edge features.

Although GNN models have successfully been applied for various molecular representation tasks, they have eluded application for carcinogenicity prediction. Motivated by this opportunity, we propose a novel system CONCERTO: Carcinogenicity prediction with GNNs. Our key contributions can be summarized as follows:

- To the best of our knowledge, we are the first to use GNN approaches and transfer learning to identify carcinogenic molecules.
- Using a novel multi-round pre-training strategy, we leverage mutagenic data to improve performance on the carcinogenicity prediction task
- We use counterfactual molecule generation to investigate known carcinogenic functional groups and validate that CONCERTO recovers biologically meaningful representations.

## 2 Related work

### 2.1 Carcinogenicity prediction

In general, molecule carcinogenicity prediction remains a challenging problem. Zhang *et al.* (2017) introduced CarcinoPred-EL, an ensemble-based approach for predicting the carcinogenicity of chemicals using molecular fingerprints, achieving a relatively high test accuracy on a limited dataset. Recently, Wang *et al.* (2020) expanded on existing methods and presented a neural network model (CapsCarcino); however, the model is not publicly available. The aforementioned methods frame carcinogenicity prediction as a classification problem, but other works attempt to predict dose-rate required for cancer onset. A number of approaches use cheminformatics features [such as molecular descriptors (Landrum, 2016; Moriwaki *et al.*, 2018)] to construct regression models for continuous carcinogenicity prediction (Fjodorova *et al.*, 2010; Singh *et al.*, 2013; Wu *et al.*, 2015). Recently, Limbu and Dakshanamurthy (2021) compared various regression models of these sorts, of which AdaBoost (Freund and Schapire, 1997) was found to be among the most accurate for dose-rate prediction.

### 2.2 Geometric deep learning

GNN models are a family of neural networks that are suited to graph structured input data and can enforce notions of permutation invariance and equivariance (Gori *et al.*, 2005; Scarselli *et al.*, 2009). Recent advancements in geometric deep learning have demonstrated success in molecular property prediction (Duvenaud *et al.*, 2015; Gilmer *et al.*, 2017; Yang *et al.*, 2019) and drug discovery (Stokes *et al.*, 2020). Duvenaud *et al.* (2015) showed utility of graph convolutional network (GCN) as an alternative way of representing a molecular profile, analogous to molecular fingerprints. In a subsequent work, Gilmer *et al.* (2017) proposed message passing neural networks (MPNNs) to predict quantum properties of organic molecular compounds, later improved upon and extended by Yang *et al.* (2019). In addition, Stokes *et al.* (2020) have demonstrated the promising outcomes of MPNN models in the domain of drug discovery, uncovering the previously unknown antimicrobial molecular compound *Halicin*. Recently, Ying *et al.* (2021) have introduced an effective position encoding technique for graph transformer architectures, finding success in molecular problem domains.

## 3 Materials and methods

### 3.1 Problem formulation

At its core, carcinogenicity prediction is a graph level regression problem: given a molecular representation, the model predicts an associated carcinogenicity dose dependent potency. We consider two types of molecular representations. The first are molecular graph representations, where the atoms and bonds of the molecule are represented as nodes and edges of the graph (respectively). The node and edge features depend on the choice of model, but include important chemical properties like atomic number, atomic mass and bond order. The second, fingerprint-based models use hand engineered features that aim to summarize important molecular properties like molecule aromaticity, presence of functional groups and atom co-occurrence (Landrum, 2016; Rogers and Hahn, 2010). Fingerprints are an effective way to incorporate domain expert knowledge through identification of important molecular substructures.

Fundamental technical challenges at the core of the carcinogenicity prediction problem are the difficulty of data acquisition, and label representation. Every experiment is conducted in animals, usually rodents, resulting in dozens of animals required for a single data point. Not only does this result in limited training data (approximately 1000 unique compounds), but presents difficulties for robust model evaluation. Previous methods have treated this problem as binary classification task which results in loss of information regarding the degree of compound potency (Doe *et al.*, 2019). This information is important for real world applications where degree of exposure is an important factor for measuring risk. Carcinogen dosing is modulated using the maximum tolerated dose (MTD) where animals are given the highest possible compound dose without compromising animal survival. The resulting datasets contain carcinogenic labeled molecules which span multiple orders of magnitude potency dosage. An ideal model for carcinogenicity prediction would be able to distinguish between dosage extremes. In certain experiments, it is possible to express results through a dose-rate formulation represented by TD50 mg/kg body weight/day, which captures the differences of compound dosage as a proportion of body weight.

### 3.2 CONCERTO

**Fig. 1.**
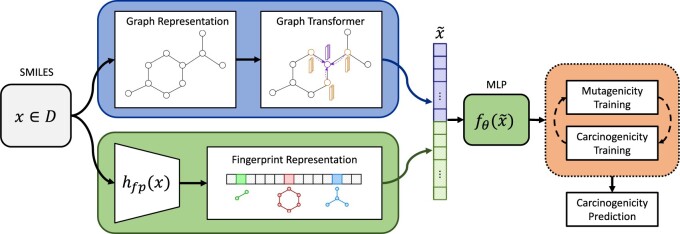
A graphical summary of the core CONCERTO components. In blue (top) is the GNN transformer, which takes in the graph representation of the molecule. In green (bottom) is the predictor which consists of fingerprint representation of the molecule that is fed into the multilayer perceptron along with the GNN representation. The two parts are jointly optimized with multi-round pre-training (orange - right) to generate carcinogenicity prediction (A color version of this figure appears in the online version of this article)

The CONCERTO architecture consists of a large self-supervised GNN transformer and multilayer perceptron (MLP) optimized over a molecular fingerprint representation concatenated with GNN transformer representation (Fig. 1). It is trained alternately on mutagenicity and carcinogenicity objectives.

#### 3.2.1 Graph neural net transformer

Given of a set of nodes (or vertices) $V={\left\{v_{i}\right\}}_{i=1}^{n}$ and a corresponding set of edges $E=\{(v_{i},v_{j})|v_{i},v_{j}\in V,i,j=1,\ldots ,n\}$, a graph is defined as a tuple G=(V,E) of the respective node and edge sets. The graph neural net transformer is composed of two components: a GNN, specifically a message passing neural network (MPNN), to encode local information about each node’s neighborhood, and a multi-head attention network (Transformer) to transmit long-range information between nodes.

GNNs take the graph structure *G* as input in the form of an adjacency matrix and use node-wise and/or edge-wise layer embeddings to learn a non-linear predictive mapping. MPNNs are a specific type of GNN which aggregate information in the form of ‘messages’ across neighborhoods of respective nodes (Gilmer *et al.*, 2017). For a one-hop neighborhood, the update for the *i*th node’s hidden state is

(1) $$h_{i}^{\left(l\right)}=\sigma (W^{\left(l\right)}m_{i}^{\left(l\right)}+b^{\left(l\right)}),$$

where $W^{\left(l\right)}$ are the neural network weights, $b^{\left(l\right)}$ is the bias term, σ(·) is some non-linear activation function, $m_{i}^{\left(l\right)}$ is an aggregation of inbound messages for the *i*th node, and l=1…L is the message passing iteration number.

Transformers use an attention mechanism to learn relationships between parts of the input data. Given a set of items, the transformer’s attention layer computes three kinds of embeddings for each item: keys, queries and values. Intuitively, the key and query embeddings are used to compare pairs of items by computing attention weights, a representation of their dependence. An item’s updated state is computed using an attention-weighted average of the value embeddings of the other items in the set. In the context of molecular property prediction, each item corresponds to an atom in the graph, and the queries, keys and values are derived from the final node embeddings of the MPNN. More formally, given a set of input queries, keys and values $\left\{(q_{k},k_{k},v_{k})\right\}$ corresponding to nodes indexed by *k*, the scaled-dot-product attention operation for node *i* is defined as

(2) $$A(i)=\sum_{j}{\left[{\text{softmax}}_{j\prime }\left(\frac{q_{i}^{T}k_{j\prime }}{\sqrt{d}}\right)\right]}_{j}v_{j},$$

where *d* is the dimensionality of the embeddings. Since $q_{k},k_{k},v_{k}$ are derived from the final MPNN node embeddings $h_{k}^{\left(L\right)}$, the attention operation does not rely on edges of the graph. Effectively, it increases the contextual node-wise neighborhood used for encoding molecular representations by allowing for interactions between nodes that are not explicitly connected.

For each molecule, we generate a representation using graph representation from self-supervised GNN transformer (GROVER) (Rong *et al.*, 2020). It is a large model that is pre-trained on 10 million unlabeled molecules utilizing self-supervised contextual and graph level motif prediction tasks. Contextual property prediction task consists of selecting a molecular subgraph and using its representation to predict local neighborhood properties. The motif prediction task involves an alternative way to encode fingerprint representations in a structure: the task consists of predicting the presence of a functional group given the entire molecular representation. Self-supervised training is conducted over representations of nodes and edges, making use of the attention operation to aggregate information across global neighborhoods. During CONCERTO training, we freeze GROVER weights and use the computed representation as input to the MLP (Rong *et al.*, 2020).

#### 3.2.2 Multilayer perceptron fingerprint predictor

To add explicit structure information, we supplement the learned representation from the graph transformer with chemical fingerprint features. We encode each molecule using Morgan, RDKit and MACCS fingerprints to capture properties relating to molecule substructures, including aromatic rings and functional groups (Durant *et al.*, 2002; Landrum, 2016; Rogers and Hahn, 2010). We concatenate the representation from GROVER to the fingerprints and train a multilayer perceptron to predict molecular carcinogenicity. We utilize ReLU activations and batch normalization to stabilize training.

#### 3.2.3 Multi-round mutagenicity pre-training

To improve carcinogenicity model predictions, we pre-train on related, more abundant mutagenicity experiments using multi-round pre-training. Instead of measuring tumor growth in animal systems to evaluate carcinogenicity, mutagenicity experiments measure compound DNA reactivity in cellular systems. DNA damage is usually evaluated with an indirect phenotypic measure (such as cell growth) resulting in noisy measurements with lower rates of reproducibility (Walmsley and Billinton, 2011). Although of lower quality, mutagenicity experiments are an order of magnitude more abundant and measure a related property to carcinogenicity experiments. To perform multi-round pre-training, we first train the model on mutagenicity data and terminate using early stopping. We then train on carcinogenicity data and perform this cycle three times, at which point we observe that the performance gains saturate. We find that multi-round pre-training increases model performance for carcinogenicity prediction. We hypothesize the effectiveness of this procedure is due to continuous cycling between objectives leading to our model learning relevant biological signal shared between tasks while ignoring irrelevant experimental noise due to neural network property of catastrophic forgetting (French, 1999)

For mutagenicity pre-training and evaluation we use a dataset generated by Hansen *et al.* (2009) consisting of 6000 unique molecules (Table 1). There is a significant overlap between mutagenicity and carcinogenicity datasets, but limited concordance: only 70% (For the purposes of this analysis we binarized carcinogenicity data.). The high mismatch percentage is in part due to low reproducibility of mutagenicity experiments (Benigni and Bossa, 2011; Walmsley and Billinton, 2011).

**Table 1.** Summary statistics of chemical compound carcinogenicity datasets

Dataset	Experiment type	No. of Experiments	(+) labels	(-) labels
CPDB	C	6540	509	494
CCRIS	C + M	88056	2674	2099
Hansen	M	N/A	3403	2909

*Note*: Under *Experiment Type*: C stands for carcinogenic experiments, M stands for mutagenic experiments. A significant fraction of compounds is present in multiple databases.

#### 3.2.4 Hyper-parameters and model selection

We perform a hyper-parameter sweep over model architecture features, training parameters and pre-training parameters. For each set of hyperparameters we perform threefold cross validation and rank the models based on the average validation performance of the folds. We then choose the top three performing models, average their prediction and evaluate on the test sets. We use canonicalized SMILES strings to identify and remove common structures, preventing data leakage across splits (Landrum, 2016; Weininger, 1988). Missing this crucial step can lead to an overlap between training and validation sets resulting in inflated performance (Li *et al.*, 2021). Our final CONCERTO model consists of a GROVER_large_ embedding in addition to 2048 dimensional Morgan, RDKit and MACCS fingerprints. The MLP consists of five layers each containing dropout with probability 0.1, batch normalization and a ReLU activation function (Agarap, 2018; Ioffe and Szegedy, 2015).

### 3.3 Counterfactual approach to model interpretability

Exmol (Wellawatte *et al.*, 2022) is a model-agnostic method for interpreting chemical property predictions. To investigate a particular prediction, Exmol creates a local chemical subspace around the target molecule and searches for nearby counterfactual examples. The subspace is generated by randomly mutating the SELFIES (Krenn *et al.*, 2019) string representation of the target (Nigam *et al.*, 2021). After applying the model to each molecule in the subspace, Exmol can find examples that are chemically close to the target with drastically different predictions. These counterfactual molecules can help explain the model’s behavior by highlighting differences in the input (i.e. functional groups, rings) that have a large effect on the output.

## 4 Experimental design

In this work, we consider a selection of chemical compound databases comprised of long-term carcinogenesis bioassays in animals, as well as short term mutagenicity experiments in bacterial cultures. To tackle the MTD problem, we augment our training set and use transformed TD50 values instead. In addition, we assemble a new external test set for evaluating carcinogenicity—five times larger than the previous (Benigni *et al.*, 2008). To confirm the validity of the new carcinogenicity test set, we evaluate the distances between molecular distributions using Tanimoto scores.

### 4.1 Continuous carcinogenicity measure

For model training we utilize CPDB, which is a collection of 6540 experimental tests containing results from long-term carcinogenesis bioassays, primarily in rodents, for over 1000 chemical compounds (Table 1) (Gold *et al.*, 2005). Carcinogenicity of the tested chemical compounds was determined using TD50 values, an estimated numerical measure of carcinogenic potency, which represent the dose-rate of tumor development. Instead of using binarized tumor growth labels, we use log reciprocal TD50 values for model training, yielding a richer information label. TD50 is estimated using the proportional hazards model (Bailer and Portier, 1993; Cox, 1972):

(3) $$\lambda (t,d)=(1+\beta *d)\lambda_{0}(t),$$

where $\lambda_{0}(t)$ is the tumor incidence without dosing (baseline) after *t* units of time, *β* is the model parameter used to calculate the TD50 value (*β *= 0 when there is no relationship between molecular dosage and tumor growth), and *d* is the administered dose-rate of a respective test molecule (Gold, 2007). The estimated TD50 value is then calculated as $\text{log}(2)/\hat{\beta }$, where $\hat{\beta }$ is the maximum likelihood estimate of *β*. We transform the calculation for TD50 to $\text{log}\;\hat{\beta }$ to improve numerical stability in model training. In addition, to summarize data across multiple experiments we take the harmonic mean over TD50 values, which biases the results toward low values, i.e. experiments that demonstrated molecular carcinogenicity. Our reasoning follows that of the CPDB authors’: given that a single experiment demonstrated carcinogenicity, the compound is likely to have some carcinogenic properties that are present in a unique set of conditions.

### 4.2 External test set

For external test evaluation we use CCRIS, which is a database containing experimental test results of over 4500 chemical compounds gathered from various studies cited in literature (Cameron *et al.*, 1986). These experiments were conducted on chronic cancer animal models, the majority of which were rodents, measuring carcinogenicity, mutagenicity, tumor promotion and tumor inhibition. A panel of experts used the aggregated experimental results to assess the molecular carcinogenic and mutagenic labels, providing binary labels for every experiment.

### 4.3 Estimating differences of molecular distributions

In this work, we introduce a new dataset for carcinogenicity analysis and use a perturbation approach for evaluating functional group importance both of which require measuring molecular distances. To measure similarity between individual molecules, we compute Tanimoto similarity of their molecular fingerprints. This pairwise measure allows us to estimate dataset variance, using the diversity metric, and distance between dataset distributions, using maximum mean discrepancy (MMD; Gretton *et al.*, 2012).

To calculate Tanimoto similarity, between two molecules $\tilde{x},\tilde{y}\in D$, we first compute binary Morgan fingerprints (Rogers and Hahn, 2010) as $x=h_{fp}(\tilde{x}),\;y=h_{fp}(\tilde{y})$, where *h_fp_* is a mapping from SMILES strings to vectorized binary representations. Then, we define the Tanimoto similarity coefficient as

(4) $$T(x,y)=\frac{\langle x,y\rangle }{||x|{|}_{2}^{2}+||y|{|}_{2}^{2}-\langle x,y\rangle },$$

where 〈·,·〉 is the vector dot product and $||\cdot |{|}_{2}$ the Euclidean norm (Maggiora *et al.*, 2014).

Since ***x*** and ***y*** are binary vectors, Equation (4) is consistent with the general definition of the Tanimoto similarity coefficient (Maggiora *et al.*, 2014).

We make use of the MMD score to define a distance metric between molecular datasets. Given two sets of molecular fingerprints that are sampled from two distributions ${\left\{x_{i}\right\}}_{i=1}^{n}∼P_{X}$ and ${\left\{y_{i}\right\}}_{i=1}^{m}∼P_{Y}$ and for some similarity kernel function $k(x_{i},y_{i})$, the empirical estimate for MMD is defined as

(5) $$\begin{array}{c} \hat{M}(P_{X},P_{Y})=\frac{1}{n(n-1)}\sum_{i}^{n}\sum_{j\neq i}^{n}k(x_{i},x_{j})\\ -\frac{2}{nm}\sum_{i}^{n}\sum_{j}^{m}k(x_{i},y_{j})+\frac{1}{m(m-1)}\sum_{i}^{m}\sum_{j\neq i}^{m}k(y_{i},y_{j}). \end{array}$$

We use the Tanimoto similarity coefficient as the kernel function, i.e. $k(x_{i},y_{i})=T(x_{i},y_{j})$.

## 5 Results

### 5.1 Model results

**Fig. 2.**
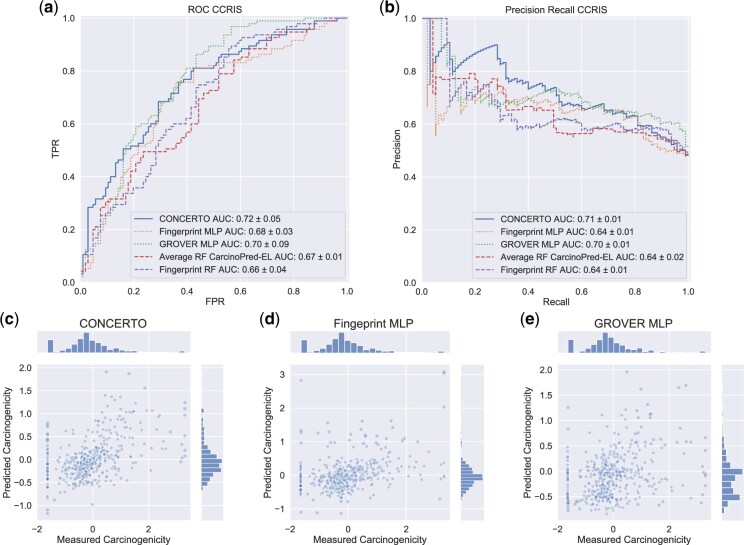
(**a**, **b**) ROC and Precision–Recall plots demonstrating performance gains of CONCERTO (solid) over previous state of the art (dashed) on an external test dataset, CCRIS. (**c–e**) Correlation between log reciprocal TD50 values and model predictions on the CPDB test set. A clustered set of points at −1.62 carcinogenicity values indicates experiments in which no tumor growth was observed in animals

We perform hyperparameter searches over CONCERTO architectures, fingerprint MLP and GROVER MLP, and find that CONCERTO outperforms the standalone models on both test sets (Table 2). Standalone GROVER results in high variability predictions underscoring the importance of the MLP-fingerprint model component. Importantly, we observe that CONCERTO outperforms other models in the low false positive region, the prediction regime in which the carcinogenicity compounds can be identified with the lowest false discovery rate (Fig. 2a). Previous state-of-the-art, CarcinoPred-EL, was trained on data from CPDB, therefore we are unable to generate predictions without overfitting. Instead, we utilize a dataset used by CarcinoPred-EL to design a comparable method and evaluate it on the continuous CPDB data. Furthermore we design an AdaBoost decision tree model emulating methodology from the work of Limbu and Dakshanamurthy (2021). We also compare on an external CCRIS test set where our model outperforms all variants of CarcinoPred-EL the AdaBoost decision tree (Table 2). We find that MLP-fingerprint predictor in conjunction with GROVER deliver the best results on the CPDB test set. We hypothesize that in data constrained settings, fingerprints are an effective way for representing domain expert knowledge.

**Table 2.** Model performances on CPDB and CCRIS

Model	CPDB *n* = 518	CPDB *n* = 518	CCRIS *n* = 202	CCRIS *n* = 202
	Pearson	MSE	ROC AUC	PR AUC
CONCERTO	0.50±0.04	0.71±0.06	0.73±0.03	0.72±0.01
Fingerprint MLP	0.36 ± 0.07	0.81 ± 0.08	0.68±0.03 (0.11)	0.64 ± 0.01
GROVER MLP	0.15 ± 0.16	0.83 ± 0.07	0.68±0.10 (0.80)	0.69 ± 0.01
CarcinoPred-EL Average RF	—	—	0.67±0.02 (0.05)	0.65 ± 0.02
CarcinoPred-EL Pubchem RF	—	—	0.64±0.03 (0.01)	0.61 ± 0.01
Fingerprint RF—CarcinoPred-EL alike	0.35 ± 0.04	1.17 ± 0.06	0.66±0.04 (0.06)	0.64 ± 0.01
Fingerprint AdaBoost—Limbu *et al.* alike	38±0.05	0.8 ± 0.08	0.68±0.02 (0.10)	0.65 ± 0.01

*Note*: ROC and PR values accompany plots a, b from Figure 2 and are calculated only over values for which CarcinoPred-EL is defined for. CarcinoPred-EL was trained on CPDB so we are unable to generate predictions without confounding overfitting. Instead, we use CarcinoPred-EL dataset to train a random forest similar to their proposed method and use it to evaluate its performance on the CPDB dataset. Uncertainty is calculated using standard deviation over data re-sampled with replacement (bootstrapping). We use one sided DeLong test to assess statistical significance differences of ROC AUC values and indicate *P*-value in parentheses. Standard deviations are indicated after the values as a measure of uncertainty. For ROC AUC significance values are indicated in paranthesis comparing to full CONCERTO model (0.73 ROC auc) were represented in Bold

### 5.2 Ablation experiments

The full CONCERTO model consists of GROVER embeddings, fingerprint representations, trained using alternating carcinogenicity and mutagenicity objectives. We perform ablation experiments to identify contributions of individual components. We conduct 50 runs with matched seeds on a well performing set of hyperparameters to evaluate whether addition of pre-training improves performance on CPDB and CCRIS. We find that providing GROVER representation to the models improves performance on both the test sets (Table 3). The mutagenicity pre-training objective further improves performance, almost matching the performance of the full CONCERTO model. In addition, we find that CONCERTO is sensitive to initialization and stochastic effects occurring during training. We hypothesize this behavior is especially common in the low data regime setting for training neural nets.

**Table 3.** Ablation experiments for CONCERTO models measuring the impact of GNN transformer, and multi-round mutagenicity pre-training

Experiment	CPDB correlation	CCRIS ROC
Fingerprint + GROVER + multi-round mutagenicity pre-training	0.37±0.10***	0.73±0.03***
Fingerprint + GROVER + mutagenicity pre-training	0.31±0.14***	0.71±0.06***
Fingerprint + GROVER	0.26±0.16***	0.68±0.09***
Fingerprint	0.17 ± 0.17	0.60 ± 0.10

*Note*: All architectures contain the MLP-fingerprint predictor. Results are averaged over 50 random seed runs. Standard deviation is computed over the random seed results. In parentheses are *P* values from a two-sided *t*-test comparing the performances from 50 models in the current cell to the cell below (***P <* *0.001).

### 5.3 Counterfactuals for model interpretability

**Fig. 3.**
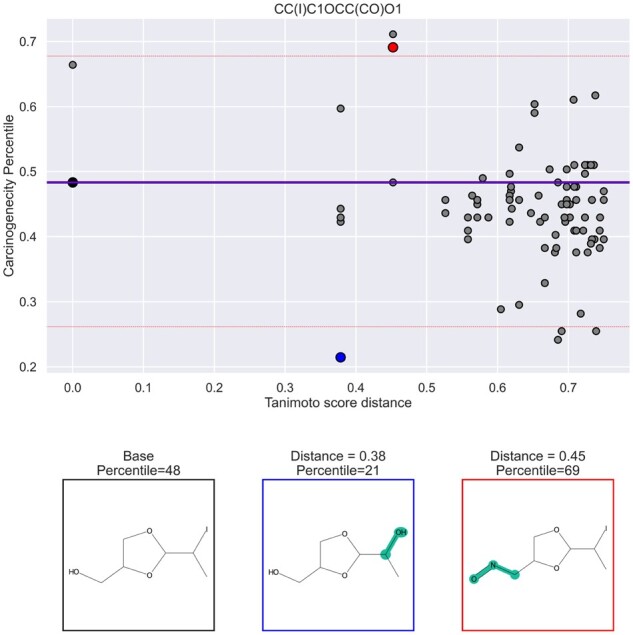
Example of a counterfactual analysis. On the *x*-axis, Tanimoto distances (1—Tanimoto similarity) are shown between sampled molecules and the original molecule. On the *y*-axis, the predicted carcinogenicity relative to test set carcinogenicity distribution is shown. For each molecule (grey point), we visualize a positive (red point) and a negative (blue point) counterfactual examples. The average within dataset diversity as measured by Tanimoto distances is 0.88. Red lines indicate prediction threshold beyond which we consider a sampled molecule a counterfactual, while blue line indicates model prediction of the base molecule (A color version of this figure appears in the online version of this article)

We utilize Exmol to better understand our model’s behavior by identifying molecular substructures that are important drivers in carcinogenicity prediction (Wellawatte *et al.*, 2022). In Figure 3, we demonstrate a method for model interpretability in which molecular substructures are added or removed from the original molecule. These changed molecules are counterfactual examples that are close to the original as measured by Tanimoto distance but have large changes in carcinogenicity predictions. In the demonstrated example, removing an aliphatic iodine leads to decreased predicted carcinogenicity. Similarly, in the increased carcinogenicity counterfactual molecule, there is addition of a nitroso group. Scientists have previously identified both of these substructures as toxicophores (Kazius *et al.*, 2005), functional groups which are enriched in mutagenic molecules. This suggests that our model is able to recover previous observations about toxicity, and by extension, carcinogenicity.

We extend the aforementioned analysis by comparing the frequencies of known toxicophores found in the test data with a corresponding set of counterfactual examples. Our results suggest that CONCERTO learns carcinogenic functional groups. For every molecule in our test set, we generate a positive carcinogenic counterfactual and a negative non-carcinogenic counterfactual. We then check the generated molecular structures for the presence of known toxicophores (Table 4) (Kazius *et al.*, 2005). We find that there is a higher percentage of toxicophores in positive counterfactuals proposed by CONCERTO than original molecules. Similarly, there is a smaller percentage of toxicophores in negative counterfactuals than original molecules. Even if a toxicophore is not present in the set of original molecules, as is the case for O[NH2], CONCERTO enriches positive counterfactuals for that functional group. Overall, this analysis is an orthogonal evaluation suggestion that CONCERTO learns individual carcinogenic functional groups.

**Table 4.** This table demonstrates the relative frequency of toxicophores in the test set and the corresponding positive and negative counterfactuals

Toxicophore	SMARTS	Substructure representation	% in negative counterfactuals	% in original molecules	% in positive counterfactuals
Nitroso	N=O	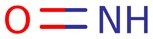	$2.75^{*}$	21.95	32.17
			(0.12)	(1.0)	(1.46)
Aliphatic halide	ClA, BrA, IA	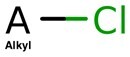	11.93	13.82	18.26
			(0.86)	(1.0)	(1.32)
Aromatic nitro	O=[N+]([O−])a	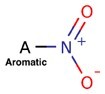	$1.83^{*}$	10.57	7.83
			(0.17)	(1.0)	(0.74)
Aromatic amine	[NH2]a	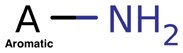	3.67	4.88	10.43
			(0.75)	(1.0)	(2.1)
Three-membered heterocycle	C1C[NH]1, C1CO1, C1CS1	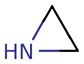	0.00	0.81	5.22
			(0.0)	(1.0)	(6.4)
Azo-type	N = N	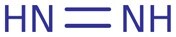	0.00	0.81	1.74
			(0.0)	(1.0)	(2.14)
Unsubsituted heteroatom-bonded heteroatom	N[NH2], N[OH], O[OH], O[NH2]	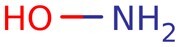	0.0	0.0	$6.96^{*}$
			(N/A)	(N/A)	(N/A)

*Note*: SMARTS are an alternative molecular string representation allowing flexible tokens for aromatic and aliphatic atoms (Landrum, 2016). In parentheses is indicated the odds ratio relative to the % of toxicophores found in original counterfactuals. Significance is calculated using fisher’s exact test over ratios of substructure matches between counterfactual and original molecules. *P* values are adjusted using Benjamini–Hochberg correction (*P <* *0.05).

### 5.4 Quantifying dataset differences

**Fig. 4.**
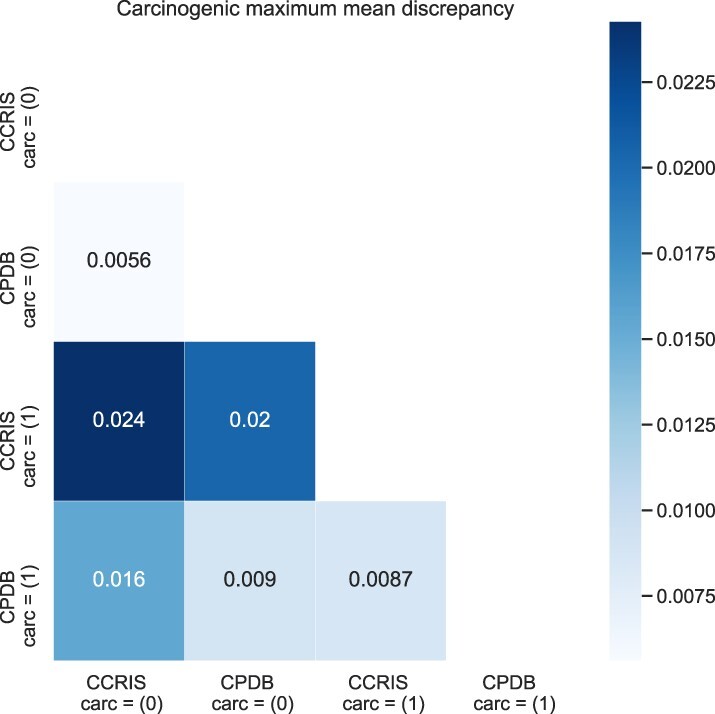
Analysis of dataset distances are generated by calculating MMD over Tanimoto scores. For carcinogenicity, there are two datasets that we further subdivide into positive and negative labels, creating four partitions. For each pair of partitions, we calculate corresponding distances and visualize using a heatmap

To better understand differences between our datasets, we calculate maximum mean discrepancy over Tanimoto scores, as described in Equations 4, 5. We perform MMD calculations for carcinogenic datasets while further partitioning data into positive and negative classes (Fig. 4). Our first observation is that as expected, the distance between matching classes across datasets is smaller than the within dataset distances between positive and negative classes. This indicates that the inter-dataset differences are smaller than inter-class differences, confirming our choice of using CCRIS as an external test dataset. Our second observation is that inter-class distances vary between different datasets. We observe that CCRIS MMD distance between positive and negative classes (0.024) is significantly greater than CPDB inter-class distance (0.009) which leads us to hypothesize about the differing nature of dataset construction. One reason for this observation could be due to the fact that CCRIS labels were assigned by a panel of experts with molecules selected at either end of the carcinogenicity spectrum. Meanwhile CPDB inclusion criteria consisted of a robust set of experimental criteria followed by the calculation of a TD50 score. These observations support our choice for utilizing CCRIS as an external test set for CPDB trained models.

## 6 Discussion

Predicting molecular carcinogenicity is an important public health problem to address, but due to the prohibitive cost and difficulty of measuring carcinogenicity many compounds lack experimental data. In this work, we investigate three orthogonal approaches to overcome dataset size constraints: architecture choice, dataset modification and pre-training techniques. First, we leverage the inductive bias present in GNN architectures relating to the graphical molecular structure. We find that GROVER, in conjunction with an MLP-fingerprint predictor outperforms the fingerprint MLP model as well as the previous state-of-the-art model. We suspect that due to the limited size of available data, a combination of transfer learning from a large GNN, and hand-engineered features extracted from molecular structures, is effective at capturing important drivers of molecular carcinogenicity. Next, we augment the dataset with more informative labels by aggregating individual experimental results and creating continuous labels. This creates a richer representation for the network and circumvents the MTD design problem, where a molecule could be carcinogenic at a maximum dose for the animal but it would be impossible to be exposed to that dosage in the natural world. In addition, we collect an external dataset five times larger than previous, allowing us to make meaningful model performance comparisons while decreasing concern of overfitting to the test set. Finally, we explore the utility of model pre-training in two forms: first utilizing transfer learning from GROVER, and second multi-round pre-training on related but lower quality experiments. The differentiable nature of our model allows us to make use of effective pre-training strategies. We assess the contributing effects of architecture choice and pre-training techniques through a series of ablation experiments (see Table 3), through which we find that mutagenicity pre-training and GROVER transfer learning are each responsible for a significant increase in performance.

Although compound carcinogenicity pre-screening is not a solved problem, we hope that CONCERTO will aid in selecting molecules for downstream carcinogenicity experiments due to its improved predictive accuracy over existing approaches. Given that up to 13% of recent drug retractions have been due to molecular DNA reactivity, a method for identifying functionally similar molecules but with decreased carcinogenicity could be useful (Onakpoya *et al.*, 2016). To that end, we demonstrate an approach for visualizing counterfactual examples (Fig. 3). We aim for this technique to be useful to domain experts for interpreting model predictions and iterating on the molecular design process. Given CONCERTO was able to identify known toxicophores, an interesting follow up direction would be to investigate novel functional groups resulting in molecular carcinogenicity by comparing counterfactuals with the corresponding original molecules.

## 7 Conclusions

In this work, we present a GNN transformer model for predicting molecule carcinogenicity. We develop a novel multi-round pre-training methodology that leverages mutagenic data to improve accuracy on the carcinogenicity task. We find that the combination of these architecture improvements and novel training techniques results in a model that outperforms the previous state-of-the-art in predicting maximum tolerable dose. Additionally, we employ counterfactual analysis to investigate model interpretability and confirm that our model recovers previous knowledge about toxicophores.
